# Supplementary material for: A Biotechnological Approach for the Production of Pharmaceutically Active Human Interferon-α from Raphanus sativus L. Plants
Source: Bioengineering (Basel). 2022 Aug 10;9(8):381. doi: 10.3390/bioengineering9080381 (PMC9404948; doi:10.3390/bioengineering9080381)
Supplement: Supplementary file 1 [file bioengineering-09-00381-s001.zip › bioengineering-1852178-supplementary.pdf]

List of Supplementary Figures

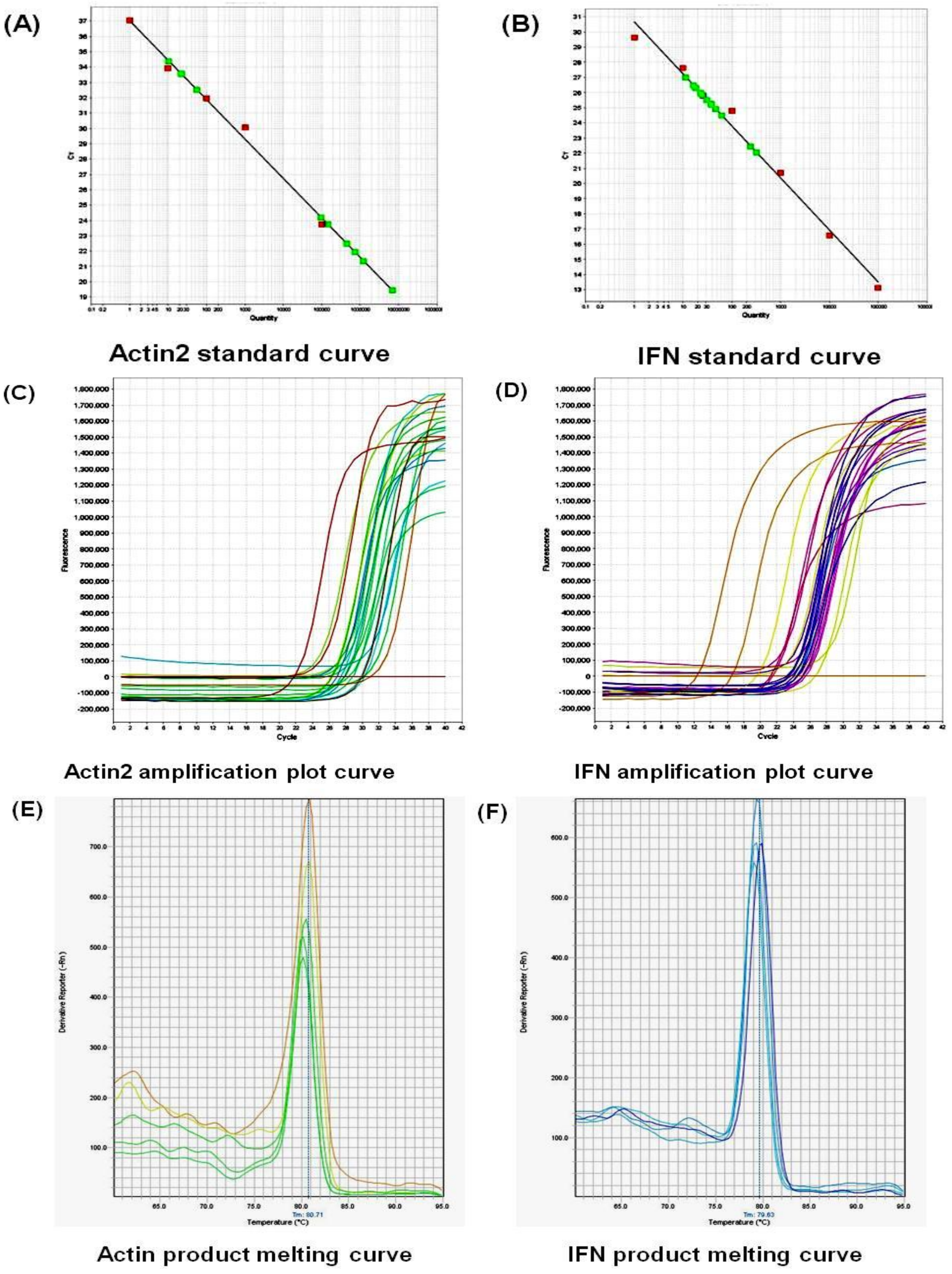

Figure S1. qRT-PCR analysis of Actin and IFNα2a RNA transcripts based on the amplification of a corresponding cDNA by StepOne qRT-PCR system (Applied biosystems, USA).

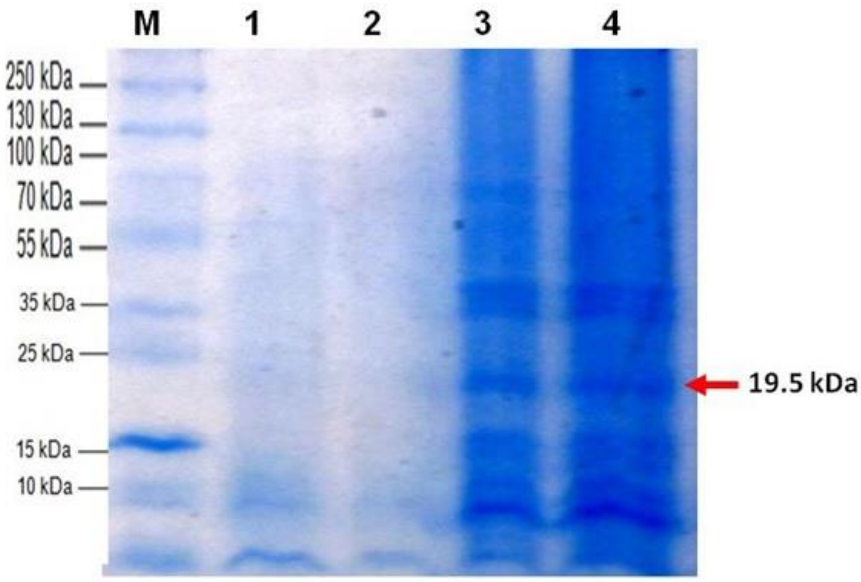

Figure S2. SDS-PAGE of the partially purified IFNα2a protein isolated from IFNα2a transgenic white and red *Raphanus sativus L* plants. M: Thermo Scientific™ PageRuler™ Unstained Protein Ladder, lane 1and 2: partially purified protein isolated from wild type white and red *Raphanus sativus L* plants. Lane 3 and 4: partially purified protein isolated from IFNα2a transgenic white and red *Raphanus sativus L* plants, respectively.

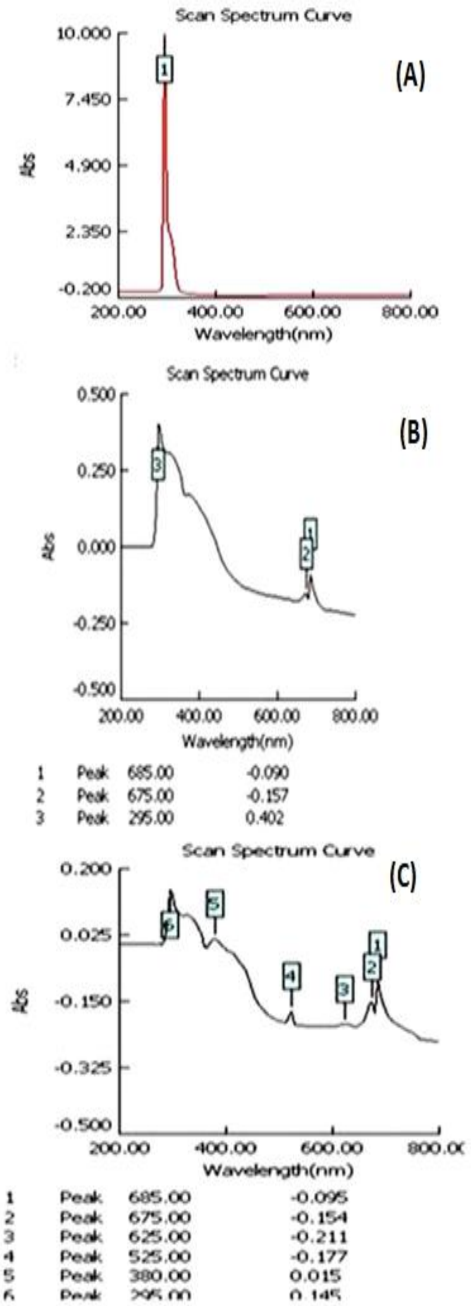

Figure S3. Scanning spectra of peg-IFN protein (A), partially purified protein fractions (B) and un-purified total protein(C) isolated from IFN.wRs-2 transgenic *Raphanus sativus L* plants.
